# The Clinical Value of the AST-to-ALT Ratio in Predicting Severity, Complications, and Prognosis in Acute Pancreatitis

**DOI:** 10.1155/grp/1922898

**Published:** 2025-11-12

**Authors:** Hao Zhang, Yao-Fei Wei, Hao-Jie Zhong, Xing-Xiang He

**Affiliations:** ^1^Department of Gastroenterology, The First Affiliated Hospital of Guangdong Pharmaceutical University, Guangzhou, China; ^2^Research Center for Engineering Techniques of Microbiota-Targeted Therapies of Guangdong Province, The First Affiliated Hospital of Guangdong Pharmaceutical University, Guangzhou, China; ^3^Department of Rheumatology, Shenzhen Traditional Chinese Medicine Hospital, The Fourth Clinical Medical College of Guangzhou University of Chinese Medicine, Shenzhen, China; ^4^Guangdong Provincial Key Laboratory for Research and Evaluation of Pharmaceutical Preparations, Guangdong Pharmaceutical University, Guangzhou, China

**Keywords:** acute pancreatitis, AST-to-ALT ratio, complications, prognosis, severity

## Abstract

**Introduction:**

The aspartate aminotransferase-to-alanine aminotransferase (AST/ALT) ratio has been widely recognized as an indicator of disease severity, complications, and prognosis in various clinical conditions. However, its relevance in acute pancreatitis (AP) has not yet been clearly established. This study is aimed at systematically evaluating the association between the AST/ALT ratio and AP, with a focus on disease severity, complication rates, and clinical outcomes.

**Methods:**

A retrospective analysis was conducted on patients diagnosed with AP at the First Affiliated Hospital of Guangdong Pharmaceutical University between July 2014 and December 2020. Multivariate logistic regression and linear regression were used to examine the relationship between the AST/ALT ratio and AP severity, complications, and prognosis.

**Results:**

A total of 207 patients were enrolled. Based on the optimal AST/ALT cut-off value determined by receiver operating characteristic curve analysis, patients were categorized into high and low AST/ALT groups. Elevated AST/ALT ratios were independently associated with severe AP, higher incidences of complications such as pleural effusion, acute heart failure, acute kidney failure, and systemic inflammatory response syndrome, as well as worse clinical outcomes, including greater vasopressor use. Linear regression further demonstrated a significant correlation between the AST/ALT ratio and severity scoring systems, including MODS, APACHE II, and Ranson scores.

**Conclusion:**

An elevated AST/ALT ratio is a strong predictor of increased disease severity, higher complication risk, and poorer prognosis in patients with AP. The AST/ALT ratio may serve as a simple, cost-effective, and sensitive biomarker for early assessment of AP progression and outcomes.

## 1. Introduction

Acute pancreatitis (AP) is a common and potentially life-threatening inflammatory disorder of the digestive system, with a global incidence estimated at 34–80 cases per 100,000 population annually [1]. Although most patients experience a mild and self-limiting course, AP can progress to severe disease accompanied by local and systemic complications, such as pancreatic necrosis, acute kidney injury, acute respiratory failure, and acute heart failure [2, 3]. Currently, the overall mortality rate of AP is approximately 1.6 per 100,000 person-years; however, in cases of acute necrotizing pancreatitis, mortality may rise to nearly 40% due to severe complications and multiple organ dysfunction [4]. Therefore, early identification of patients at risk of developing severe disease and complications is critical for improving clinical outcomes. Although several biomarkers—including serum amylase, C-reactive protein (CRP), immature granulocyte percentage, and the neutrophil-to-lymphocyte ratio—have been proposed for evaluating AP severity and prognosis, an ideal laboratory indicator with high sensitivity, specificity, and clinical practicality is still lacking [5].

The liver is among the earliest and most frequently affected extra-pancreatic organs in AP, with hepatic injury occurring in approximately 15%–60% of patients [6]. In recent years, the aspartate aminotransferase-to-alanine aminotransferase (AST/ALT) ratio has gained attention as a potential biomarker associated with disease progression and prognosis in various clinical settings. For example, Iwata et al. reported a strong positive association between the AST/ALT ratio and hepatic dysfunction, as assessed by the Child–Pugh score, in patients with liver cirrhosis [7]. Moreover, the AST/ALT ratio has been implicated in predicting postoperative outcomes in urinary and gastrointestinal malignancies, as well as in the onset and prognosis of cardiovascular diseases, including heart failure and myocardial infarction [6–9]. Additionally, elevated AST/ALT ratios have been associated with poor survival among patients with esophageal cancer [10]. Despite these findings, the clinical significance of the AST/ALT ratio in the context of AP remains largely unexplored.

Therefore, this study was aimed at systematically investigating the relationship between the AST/ALT ratio and AP, with particular emphasis on its association with disease severity, the development of complications, and clinical prognosis. By evaluating these correlations, we sought to determine whether the AST/ALT ratio could serve as a simple, cost-effective, and reliable biomarker for early risk stratification in patients with AP.

## 2. Materials and Methods

### 2.1. Participants

This retrospective study included 207 patients diagnosed with AP who were admitted to the First Affiliated Hospital of Guangdong Pharmaceutical University between July 2014 and December 2020. Patients were excluded if they met any of the following criteria: (1) a history of chronic pancreatitis, (2) preexisting chronic liver diseases, (3) pregnancy, (4) previous organ transplantation, or (5) incomplete clinical or laboratory data in the electronic medical record system.

### 2.2. Data Collection

Clinical data for all eligible patients were retrospectively retrieved from the electronic medical record system of the First Affiliated Hospital of Guangdong Pharmaceutical University. The collected information included demographic characteristics such as age and sex, as well as lifestyle factors including smoking and alcohol consumption. Medical history was recorded, with particular attention to hypertension, diabetes mellitus, and anemia. Laboratory parameters on admission were documented and encompassed white blood cell (WBC) count, red blood cell (RBC) count, platelet (PLT) count, serum amylase, lipase, alanine aminotransferase (ALT), aspartate aminotransferase (AST), total bilirubin (TBil), lactate dehydrogenase (LDH), CRP, blood urea nitrogen (BUN), and serum creatinine (SCR). In addition, information on disease-related complications, including pancreatic necrosis, pleural effusion, ascites, myocardial infarction, acute heart failure, acute kidney failure, acute respiratory failure, and systemic inflammatory response syndrome (SIRS), was collected. To assess disease severity, several validated scoring systems were used, including the Acute Physiology and Chronic Health Evaluation II (APACHE II), Bedside Index for Severity in Acute Pancreatitis (BISAP), Modified Computed Tomography Severity Index (CTSI), Multiple Organ Dysfunction Syndrome (MODS) score, and SIRS score. Data regarding treatment interventions, such as vasopressor or diuretic administration and tracheal intubation, were also recorded. Finally, clinical outcomes including hospital length of stay, total hospital costs, transfer to the intensive care unit (ICU), and in-hospital mortality were documented to evaluate prognosis.

### 2.3. Definitions

The diagnosis of AP was established according to the 2012 revised Atlanta criteria, requiring the presence of at least two of the following three features: persistent upper abdominal pain consistent with AP, serum amylase or lipase levels at least three times higher than the upper limit of normal, and characteristic imaging findings on abdominal computed tomography, magnetic resonance imaging, or ultrasonography [3]. Hypertension was defined as systolic blood pressure ≥ 130 mmHg and/or diastolic blood pressure ≥ 80 mmHg in accordance with current clinical guidelines. Pancreatic necrosis was confirmed radiologically by the presence of nonenhancing areas within the pancreatic parenchyma on contrast-enhanced CT. The definition of acute respiratory failure [11], pleural effusion [12], acute heart failure [13], myocardial infarction [14], acute kidney failure [15], and ascites [16] adhered to the respective guidelines. The severity of AP was assessed using the Ranson score, MODS score [17], BISAP score [18], APACHE II score [19], and modified CTSI score [20].

### 2.4. Statistical Analysis

All statistical analyses were performed using SPSS software (Version 25.0; IBM Corp., Armonk, NY, United States). The normality of continuous data was assessed using the Kolmogorov–Smirnov test. Variables with a normal distribution were expressed as mean ± standard deviation (SD), whereas nonnormally distributed variables were presented as median and interquartile range (IQR). Categorical variables were summarized as frequencies and percentages. Comparisons between two groups were conducted using independent-sample *t*-tests for normally distributed data and Mann–Whitney *U* tests for nonnormally distributed data, while the chi-square test or Fisher's exact test was applied for categorical variables, as appropriate. For comparisons involving more than two groups, one-way analysis of variance (ANOVA) was used for normally distributed data, and the Kruskal–Wallis test was applied for skewed data. To examine whether the AST/ALT ratio served as an independent risk factor for complications, interventions, and prognosis, forward stepwise binary logistic regression was employed, and results were reported as odds ratios (ORs) with 95% confidence intervals (CIs). Additionally, forward stepwise multiple linear regression was used to evaluate the association between the AST/ALT ratio and severity scores, hospital duration and hospital expenses, with results expressed as beta coefficients (*β*) and standard errors (SE). Potential confounders including age, sex, and anemia were adjusted in all regression models. A two-sided *p* value of < 0.05 was considered to indicate statistical significance.

## 3. Results

### 3.1. Patient Characteristics

A total of 207 patients diagnosed with AP were enrolled in the study and classified according to the revised Atlanta criteria into mild AP (MAP, *n* = 108), moderately severe AP (MSAP, *n* = 79), and severe AP (SAP, *n* = 20). The baseline clinical features of the three groups are summarized in Table 1.

Although no significant differences were observed in age or sex distribution among the three groups, patients with SAP exhibited markedly worse laboratory profiles. Specifically, SAP cases showed higher WBC counts and significantly elevated levels of LDH, CRP, SCR, and BUN compared to the MAP and MSAP groups (all *p* < 0.05). Conversely, RBC and PLT counts were significantly lower in patients with SAP, reflecting hemoconcentration and potential bone marrow suppression or consumption. Importantly, the median AST/ALT ratio was significantly higher in the SAP group compared with MAP and MSAP patients (*p* = 0.007), suggesting its potential association with disease severity.

### 3.2. ROC Curve Analysis of AST/ALT for Predicting SAP

**Figure 1:**
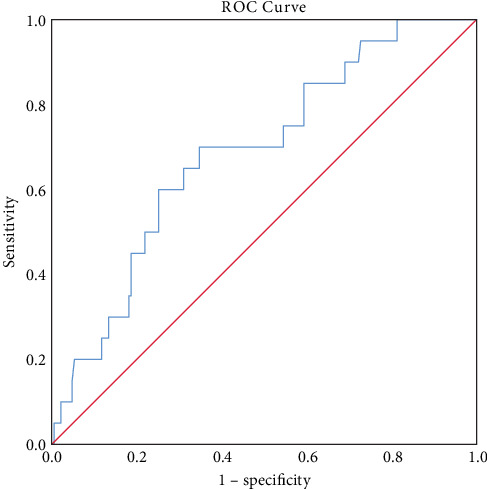
ROC curve of AST/ALT ratio for predicting severe acute pancreatitis. AUC = 0.686 (95% CI: 0.570–0.802); optimal cut-off = 1.496.

The predictive ability of the AST/ALT ratio for identifying severe AP was evaluated using ROC curve analysis (Figure 1). The SAP group was defined as the positive outcome and the MAP and MSAP groups as the negative outcome. The AST/ALT ratio yielded an AUC of 0.686 (95% CI: 0.570–0.802, *p* = 0.006), indicating moderate discriminative performance. The optimal cut-off value determined by Youden's index was 1.496, which was subsequently used to stratify patients. This threshold provided the greatest balance between sensitivity and specificity for predicting SAP.

### 3.3. Baseline Characteristics Stratified by the Optimal AST/ALT Cut-Off

Based on the cut-off value of 1.496, patients were divided into a low AST/ALT group (< 1.496, *n* = 128) and a high AST/ALT group (≥ 1.496, *n* = 79). The baseline characteristics of the two groups are presented in Table 2.

Demographic characteristics, including age, smoking status, and alcohol consumption, were comparable between the groups. However, the proportion of male patients was significantly higher in the low AST/ALT group (59.38% vs. 43.04%, *p* = 0.031). Etiologically, gallstone-related pancreatitis was more frequent in the AST/ALT < 1.496 group (56.30% vs. 35.40%), whereas idiopathic pancreatitis was more prevalent in patients with AST/ALT ≥ 1.496 (50.60% vs. 25.80%) (*p* = 0.009). No significant differences were observed regarding hypertension, diabetes mellitus, or other comorbidities.

### 3.4. Laboratory Parameters and Disease Severity Scores

Patients with an AST/ALT ≥ 1.496 showed more adverse biochemical profiles than those with AST/ALT < 1.496. They had significantly lower RBC, PLT, ALT, and TBil levels, but higher LDH and BUN (Table 3), indicating greater hepatic injury, metabolic stress, and renal dysfunction. Correlation analysis further confirmed that the AST/ALT ratio was negatively associated with RBC (*r* = −0.326), PLT (*r* = −0.152), ALT (*r* = −0.516), and TBil (*r* = −0.231) and positively correlated with LDH (*r* = 0.281), SCR (*r* = 0.141), and BUN (*r* = 0.304) (all *p* < 0.05; Table 4).

Consistently, patients with elevated AST/ALT ratios had significantly higher MODS, BISAP, APACHE II, and Ranson scores (Table 5), suggesting a close association between high AST/ALT and increased systemic inflammatory burden and disease severity in AP.

### 3.5. Complications

Patients with an AST/ALT ≥ 1.496 experienced significantly higher rates of major complications compared with those with AST/ALT < 1.496. Specifically, the high AST/ALT group had increased incidences of acute heart failure (10.13% vs. 2.34%; *p* = 0.023), myocardial infarction (6.33% vs. 0.78%; *p* = 0.031), acute kidney failure (24.05% vs. 5.47%; *p* < 0.001), and SIRS (40.51% vs. 26.56%; *p* = 0.046).

Although pleural effusion and acute respiratory failure were more frequent in the higher AST/ALT group, these differences did not reach statistical significance (Table 5).

### 3.6. Interventions and Prognosis

Patients with an AST/ALT ≥ 1.496 required more intensive clinical interventions. Specifically, the use of vasopressors (10.13% vs. 1.56%; *p* = 0.008) and diuretics (24.05% vs. 12.50%; *p* = 0.037) was significantly higher in this group compared with patients with AST/ALT < 1.496. In terms of clinical outcomes, the high AST/ALT group also had a significantly higher in-hospital mortality rate (7.59% vs. 0%; *p* = 0.003). In contrast, no significant differences were observed between the two groups in hospital stay duration, hospitalization costs, ICU transfer rate, or incidence of tracheal intubation, the latter occurring in only one patient in each group (Table 6).

### 3.7. Binary Logistic Regression Analyses

Binary logistic regression was conducted to evaluate whether an elevated AST/ALT ratio independently predicted severe AP, related complications, and adverse outcomes. After adjusting for potential confounders, a high AST/ALT ratio was identified as an independent risk factor for pleural effusion (OR = 2.039; 95% CI: 1.141–3.645; *p* = 0.013), acute heart failure (OR = 4.695; 95% CI: 1.207–18.260; *p* = 0.026), acute kidney failure (OR = 5.474; 95% CI: 2.181–13.738; *p* < 0.001), SIRS (OR = 1.966; 95% CI: 1.070–3.612; *p* = 0.029), and vasopressor use (OR = 7.099; 95% CI: 1.467–34.345; *p* = 0.015) (Table 7).

### 3.8. Linear Regression Analyses

Multiple linear regression was performed to further assess the association between the AST/ALT ratio and disease severity. After adjusting for potential confounders, a higher AST/ALT ratio was independently associated with increased MODS (*β* = 1.310, SE = 0.269; *p* < 0.001), APACHE II (*β* = 2.972, SE = 0.680; *p* < 0.001) and Ranson scores (*β* = 0.393, SE = 0.142; *p* = 0.006). These findings confirm that patients with an AST/ALT ≥ 1.496 had significantly higher severity scores compared with those with AST/ALT < 1.496 (Table 8).

## 4. Discussion

Conventional scoring systems such as APACHE II, BISAP, and CTSI are widely used to evaluate the severity of AP. Although these tools demonstrate high predictive accuracy in identifying severe AP (AUC values in our study: BISAP 0.93, APACHE II 0.86, and CTSI 0.73), their clinical utility is limited by the complexity of parameter collection, delayed calculation, and poor feasibility in emergency settings. Therefore, there is a strong need for simple, rapid, and cost-effective biomarkers for early risk stratification. In this context, our study provides novel evidence that the AST/ALT ratio is significantly associated with disease severity, complications, and prognosis in AP patients, highlighting its potential value as an accessible prognostic indicator.

In this study, we demonstrated for the first time that an elevated AST/ALT ratio is strongly correlated with increased disease severity, higher rates of complications, and worse short-term prognosis in patients with AP. Patients with higher AST/ALT ratios exhibited significantly increased MODS, BISAP, APACHE II, and Ranson scores, suggesting that the AST/ALT ratio reflects both systemic inflammatory burden and organ dysfunction. These findings are in line with previous research in other diseases, where the AST/ALT ratio has been reported as a useful prognostic marker in chronic heart failure, diabetic retinopathy, and various malignancies [21, 22]. Our results extend these observations to AP and indicate that the AST/ALT ratio, a routine laboratory parameter, may serve as a simple and efficient adjunct to existing scoring systems for early risk assessment.

Several mechanisms may explain the association between a high AST/ALT ratio and more severe disease in AP. AST is widely expressed in extrahepatic tissues such as the heart, skeletal muscle, and kidneys, whereas ALT is mainly confined to hepatocytes. Thus, an increased AST/ALT ratio may reflect not only hepatic dysfunction but also systemic tissue injury and multiorgan stress. Experimental studies have shown that elevated AST and ALT levels are linked to oxidative stress, mitochondrial dysfunction, and activation of systemic inflammatory pathways [23]. These processes play a critical role in the progression of AP, contributing to pancreatic necrosis, endothelial injury, and cytokine release [24]. Although direct biomarkers of oxidative stress (e.g., SOD and GSH) have higher specificity, their high cost and limited availability restrict routine clinical use [25, 26]. In contrast, the AST/ALT ratio is easily accessible and may serve as a practical surrogate marker of systemic inflammation and oxidative damage in AP.

Our study also revealed that a higher AST/ALT ratio was significantly associated with major AP-related complications, including acute kidney failure, acute heart failure, and SIRS. This association may be explained by the systemic impact of severe inflammation and ischemia-reperfusion injury in AP. Elevated AST levels have been linked to increased oxidative stress, endothelial dysfunction, and activation of matrix metalloproteinases, which together contribute to impaired cardiac microcirculation and myocardial injury, thereby increasing the risk of acute heart failure [27]. Similarly, a high AST/ALT ratio has been associated with activation of the renin–angiotensin system and elevated proinflammatory cytokines such as IL-6 and TNF-*α*, which can trigger renal vasoconstriction, reduce glomerular filtration, and promote kidney parenchymal damage [28]. These findings may account for the higher need for vasopressors and the significantly increased mortality observed in patients with elevated AST/ALT ratios in our cohort.

The prognostic value of the AST/ALT ratio has been increasingly recognized across a range of extrahepatic diseases, including cardiac arrest, ischemic stroke, and renal cell carcinoma, where higher values are associated with increased ICU admission and mortality [29–31]. Our study extends these observations to AP. Consistent with those findings, patients with elevated AST/ALT ratios in our cohort had higher rates of vasopressor use, a greater incidence of organ failure, and a significantly increased in-hospital mortality rate. These results suggest that the AST/ALT ratio reflects not only hepatic dysfunction but also systemic inflammatory injury and multiorgan impairment. Given its wide availability, low cost, and rapid acquisition, the AST/ALT ratio may serve as a practical adjunct to existing scoring systems, particularly in early triage, bedside assessment, and resource-limited settings.

Despite its strengths, including the use of multiple validated severity scores and comprehensive analysis of complications and outcomes, this study has several limitations. First, its retrospective and single-center design may introduce selection bias and limit generalizability. Second, only baseline AST/ALT values were evaluated; dynamic changes during hospitalization, which may better reflect disease progression, were not assessed. Third, although multivariate adjustments were performed, residual confounding factors—such as variations in time from symptom onset to hospital admission or prior medication use—cannot be fully excluded. Future multicenter prospective studies with larger sample sizes and serial monitoring of AST/ALT are warranted to validate our findings. Additionally, mechanistic research is needed to clarify whether the AST/ALT ratio is merely a biomarker or actively involved in the pathophysiology of organ injury in AP.

## 5. Conclusion

Elevated AST/ALT ratio was independently associated with greater disease severity, higher complication rates, and increased mortality in AP. As a simple, inexpensive and widely available biomarker, it may assist in early risk stratification and timely clinical intervention. Further prospective and mechanistic studies are warranted to validate its predictive value and clarify its pathophysiological relevance.

## Figures and Tables

**Table 1:** Baseline characteristics of patients with acute pancreatitis stratified by severity.

	**MAP **(**n** = 108)	**MSAP **(**n** = 79)	**SAP **(**n** = 20)	**p** ** value**
Age (years)	63.00 (46.00–81.00)	66.00 (48.00–81.00)	74.00 (55.25–85.00)	0.244
Male sex (%)	54 (50.00)	47 (59.50)	9 (45.00)	0.326
WBC (×10^9^/L)	8.17 (5.81–11.80)	10.94 (7.94–14.15)	11.70 (7.62–13.25)	0.015
RBC (×10^12^/L)	4.07 (3.69–4.58)	4.21 (3.43–4.69)	3.22 (2.20–4.58)	0.043
PLT (×10^9^/L)	213.50 ± 86.49	224.90 ± 89.83	155.30 ± 80.28	0.007
Amylase (U/L)	340.50 (173.30–879.00)	438.50 (197.80–1061.00) (*n* = 78)	408.50 (232.50–883.30)	0.492
Lipase (U/L)	337.10 (95.50–736.80) (*n* = 103)	449.40 (156.40–824.50) (*n* = 74)	364.60 (256.90–673.70)	0.395
ALT (U/L)	34.00 (16.00–153.30)	35.00 (17.00–157.00)	24.00 (13.50–189.30)	0.924
AST (U/L)	46.00 (23.25–134.80)	38.00 (24.00–110.00)	63.00 (35.25–373.30)	0.185
TBil (*μ*mol/L)	15.00 (9.60–31.50) (*n* = 107)	16.70 (11.50–37.5)	23.70 (7.15–62.30)	0.366
LDH (*μ*mol/L)	236.00 (188.00–363.00) (*n* = 85)	232.00 (187.80–313.80) (*n* = 66)	478.00 (277.50–710.50)	0.001
CRP (U/L)	20.57 (9.10–72.08) (*n* = 84)	30.75 (12.30–96.03) (*n* = 58)	95.38 (39.70–183.80) (*n* = 18)	0.008
SCR (*μ*mol/L)	72.50 (58.25–95.50) (*n* = 104)	68.00 (56.00–98.00)	167.00 (77.00–304.80)	0.001
BUN (mmol/L)	5.43 (3.98–7.35) (*n* = 104)	5.55 (4.28–7.67)	15.86 (7.23–23.30)	< 0.001
AST/ALT	1.27 (0.81–2.10)	1.14 (0.63–1.67)	1.88 (1.09–2.88)	0.007

*Note:* Data are presented as mean ± standard deviation (SD), medians (interquartile ranges) or *n* (%).

Abbreviations: ALT, alanine aminotransferase; AST, aspartate transaminase; BUN, blood urea nitrogen; CRP, C-reactive protein; LDH, lactate dehydrogenase; PLT, platelet; RBC, red blood cells; SCR, serum creatinine; TBil, total bilirubin; WBC, white blood cells.

**Table 2:** Characteristics between patients below or above the best cut-off value of AST/ALT ratio.

	**A** **S** **T**/**ALT** < 1.496 **(n** = 128**)**	**A** **S** **T**/**ALT**≧1.496 **(n** = 79**)**	**p** ** value**
Age (years)	64.50 (47.00–80.00)	68.00 (50.00–83.00)	0.265
Male sex (%)	76 (59.38)	34 (43.04)	0.031
Alcoholism (%)	10 (7.81)	3 (3.80)	0.378
Smoker (%)	16 (12.50)	8 (10.13)	0.661
Hypertension (%)	43 (33.59)	32 (40.51)	0.372
Diabetes (%)	23 (17.97)	18 (22.78)	0.473
Etiology (%)			0.009
Gallstones	72 (56.30)	28 (35.40)	
Hypertriglyceridemia	16 (12.50)	5 (6.30)	
Alcohol	1 (0.80)	1 (1.30)	
Post-ERCP	3 (2.30)	3 (3.80)	
Medications	3 (2.30)	2 (2.50)	
Idiopathic	33 (25.80)	40 (50.60)	

*Note:* Data are presented as medians (interquartile ranges) or *n* (%).

Abbreviations: ALT, alanine aminotransferase; AST, aspartate transaminase.

**Table 3:** Laboratory parameters between patients below or above the best cut-off value of AST/ALT ratio.

	**A** **S** **T**/**ALT** < 1.496 **(n** = 128**)**	**A** **S** **T**/**ALT**≧1.496 **(n** = 79**)**	**p** ** value**
WBC (×10^9^/L)	10.19 (6.24–13.04)	9.28 (6.56–12.68)	0.735
RBC (×10^12^/L)	4.22 (3.72–4.79)	3.71 (2.82–4.41)	< 0.001
PLT (×10^9^/L)	215.50 (169.00–268.50)	191.00 (124.00–259.00)	0.027
Amylase (U/L)	449.00 (208.00–1042.00) (*n* = 127)	344.00 (165.00–831.00)	0.238
Lipase (U/L)	439.40 (149.10–782.20) (*n* = 120)	302.80 (102.70–698.60) (*n* = 77)	0.141
ALT (U/L)	65.50 (23.001–217.80)	16.00 (9.00–92.00)	< 0.001
AST (U/L)	48.00 (24.00–126.50)	40.00 (24.00–165.00)	0.694
TBil (*μ*mol/L)	17.40 (11.70–36.10) (*n* = 127)	13.00 (8.30–30.80)	0.024
LDH (*μ*mol/L)	220.00 (179.00–303.00) (*n* = 103)	301.50 (211.30–642.50) (*n* = 68)	< 0.001
CRP (U/L)	20.60 (8.60–82.95) (*n* = 98)	44.30 (18.73–102.90) (*n* = 62)	0.067
SCR (*μ*mol/L)	71.50 (59.00–90.00) (*n* = 124)	82.00 (56.00–172.00)	0.104
BUN (mmol/L)	5.43 (4.08–6.91) (*n* = 124)	6.97 (4.10–16.87)	0.002

*Note:* Data are presented as medians (interquartile ranges) or *n* (%).

Abbreviations: ALT, alanine aminotransferase; AST, aspartate transaminase; BUN, blood urea nitrogen; CRP, C-reactive protein; LDH, lactate dehydrogenase; PLT, platelet; RBC, red blood cells; SCR, serum creatinine; TBil, total bilirubin; WBC, white blood cells.

**Table 4:** Spearman correlation analysis between AST/ALT ratio and laboratory parameters.

**Variable**	**Correlation coefficient**	**p** ** value**
WBC (×10^9^/L)	−0.036	0.606
RBC (×10^12^/L)	−0.326	< 0.001
PLT (×10^9^/L)	−0.152	0.028
Amylase (U/L)	−0.108	0.122
Lipase (U/L)	−0.071	0.321
ALT (U/L)	−0.516	< 0.001
AST (U/L)	−0.027	0.698
TBil (*μ*mol/L)	−0.231	0.001
LDH (*μ*mol/L)	0.281	< 0.001
CRP (U/L)	0.132	0.097
SCR (*μ*mol/L)	0.141	0.045
BUN (mmol/L)	0.304	< 0.001

Abbreviations: ALT, alanine aminotransferase; AST, aspartate transaminase; BUN, blood urea nitrogen; CRP, C-reactive protein; Hb, hemoglobin; LDH, lactate dehydrogenase; PLT, platelet; RBC, red blood cells; SCR, serum creatinine; TBil, total bilirubin; WBC, white blood cells.

**Table 5:** Disease severity scores and complications between patients below or above the best cut-off value of AST/ALT ratio.

	**A** **S** **T**/**ALT** < 1.496** (n** = 128**)**	**A** **S** **T**/**ALT**≧1.4961** (n** = 79**)**	**p** ** value**
Severity scores			
Modified CTSI	2.00 (2.00–4.00)	2.00 (2.00–4.00)	0.393
MODS	1.00 (0.00–2.00)	1.00 (0.00–4.00)	0.001
BISAP	1.00 (0.00–2.00)	2.00 (1.00–3.00)	< 0.001
APACHE II	7.00 (4.25–9.75)	10.00 (6.00–15.00)	< 0.001
Ranson	1.00 (1.00–2.00)	2.00 (1.00–3.00)	0.005
Complications			
Pancreatic necrosis (%)	12 (9.84)	5 (7.94)	0.792
Acute respiratory failure (%)	4 (3.13) (*n* = 122)	7 (8.86) (*n* = 63)	0.109
Pleural effusion (%)	44 (34.38)	41 (51.90)	0.014
Acute heart failure (%)	3 (2.34)	8 (10.13)	0.023
Myocardial infarction (%)	1 (0.78)	5 (6.33)	0.031
Acute kidney failure (%)	7 (5.47)	19 (24.05)	< 0.001
Ascites (%)	21 (16.41)	21 (26.58)	0.109
SIRS (%)	34 (26.56)	32 (40.51)	0.046

*Note:* Data are presented as medians (interquartile ranges) or *n* (%).

Abbreviations: APACHE, acute physiologic assessment and chronic health evaluation; BISAP, bedside index for severity in acute pancreatitis; CTSI, CT severity index; MODS, multiple organ dysfunction syndrome; SIRS, systemic inflammatory response syndrome.

**Table 6:** Interventions and prognosis between patients below or above the best cut-off value of AST/ALT ratio.

	**A** **S** **T**/**ALT** < 1.496 **(n** = 128**)**	**A** **S** **T**/**ALT**≧1.496 **(n** = 79**)**	**p** ** value**
Interventions			
Use of vasopressor (%)	2 (1.56)	8 (10.13)	0.008
Use of diuretic agent (%)	16 (12.50)	19 (24.05)	0.037
Tracheal intubation (%)	1 (0.78)	1 (1.27)	0.729
Prognosis			
Hospital duration (days)	10.00 (7.00–14.75)	11.00 (7.00–15.00)	0.598
Hospital expenses (yuan)	24,634.00 (14,637.00–45,262.00)	27,239.00 (18,286.00–45,734.00)	0.252
Transfer to ICU (%)	2 (1.56)	2 (2.53)	0.637
Mortality (%)	0	6 (7.59)	0.003

*Note:* Data are presented as median (interquartile range) or *n* (%).

Abbreviation: ICU, intensive care unit.

**Table 7:** Logistic regression analysis of high AST/ALT ratio as a risk factor for severe acute pancreatitis, complications, and poor prognosis.

	**OR (95% CI)**	**p** ** value**
Complications		
Pancreatic necrosis (%)	—	—
Acute respiratory failure (%)	—	—
Pleural effusion (%)	2.039 (1.141–3.645)	0.013
Acute heart failure (%)	4.695 (1.207–18.264)	0.026
Myocardial infarction (%)	—	—
Acute kidney failure (%)	5.474 (2.181–13.738)	< 0.001
Ascites (%)	—	—
SIRS (%)	1.966 (1.070–3.612)	0.029
Interventions and prognosis		
Use of vasopressor (%)	7.099 (1.467–34.345)	0.015
Use of diuretic agent (%)	—	—
Tracheal intubation (%)	—	—
Transfer to ICU (%)	—	—
Mortality (%)	—	—

*Note:* Data were adjusted for age, sex, and etiology.

Abbreviations: CI, confidence interval; ICU, intensive care unit; OR, odds ratio; SIRS, systemic inflammatory response syndrome.

**Table 8:** Multivariate linear regression analyses of high AST/ALT ratio as a risk factor for hospital duration, hospital expenses and acute pancreatitis severity scores.

	**Beta (** **β** **)**	**Standard error (SE)**	**p** ** value**
Hospital duration (days)	—	—	—
Hospital expenses (yuan)	—	—	—
Modified CTSI	—	—	—
MODS	1.310	0.269	< 0.001
BISAP	—	—	—
APACHE II	2.972	0.680	< 0.001
Ranson	0.393	0.142	0.006

*Note:* Data were adjusted for age, sex, and etiology.

Abbreviations: APACHE, acute physiologic assessment and chronic health evaluation; BISAP, bedside index for severity in acute pancreatitis; CTSI, CT severity index; MODS, multiple organ dysfunction syndrome.

## Data Availability

All data generated or analyzed in this study are included in this article. Further enquiries can be directed to the author Hao Zhang via e-mail (17339851668@163.com).
